# Factors associated with parental literacy and hesitancy toward pediatric vaccination

**DOI:** 10.1186/s12889-025-23410-y

**Published:** 2025-07-02

**Authors:** Yi Zheng, Paula M. Frew, Dong Wang, Amanda L. Eiden

**Affiliations:** 1 Biostatistics and Research Decision Sciences, Merck & Co., Inc, 126 East Lincoln Avenue, P.O. Box 2000, Rahway, NJ 07065 USA; 2 Global Medical and Scientific Affairs, Merck & Co., Inc, 126 East Lincoln Avenue, P.O. Box 2000, Rahway, NJ 07065 USA; 3 Outcomes Research, Merck & Co., Inc, 351 N Sumneytown Pike, North Wales, PA 19454 USA

**Keywords:** Cross-sectional studies, Immunization, Vaccine literacy, Health literacy, Vaccine hesitancy, Vaccine confidence

## Abstract

**Background:**

Vaccine literacy (knowledge about vaccines and the ability to identify accurate information about them) and vaccine hesitancy (delay in or refusal of vaccination) among parents may affect immunization decision-making and pediatric vaccine uptake. This study aimed to examine the demographic and socioeconomic characteristics of parents by vaccine literacy and hesitancy and to assess the relationship between attitudinal, behavioral, and experiential factors and parents’ vaccine literacy and hesitancy.

**Methods:**

A cross-sectional survey was administered in 2022 to US adults aged ≥ 18 (*N* = 692) who self-identified as parents/guardians of children aged < 18. Parents were asked questions about their experiences assessing information on pediatric vaccinations, their information-seeking and decision-making practices, interactions with healthcare providers (HCPs) regarding pediatric vaccines, beliefs about vaccine efficacy and safety, trust in scientific and medical information from various sources, perceptions of the danger and severity of vaccine-preventable diseases, and the benefits and convenience of vaccination. High vaccine literacy was defined as being moderately or extremely familiar with the vaccines a child should receive. Vaccine hesitancy was defined as being somewhat or very hesitant, or not sure, about childhood immunizations (i.e., shots). An exploratory factor analysis reflected 7 discrete underlying variables describing self-reported behaviors, beliefs, and experiences regarding childhood vaccination.

**Results:**

Vaccine literacy and hesitancy were associated with parents’ gender, race/ethnicity, education level, and urbanicity. Factors associated with increased vaccine literacy were positive interactions with HCPs (OR 2.24, *P* < 0.001), active information-seeking behavior (OR 1.88, *P* < 0.001), positive beliefs about vaccines (OR 1.39, *P* = 0.001), and beliefs about vaccination requirements (OR 1.21, *P* = 0.041). Trouble understanding information from HCPs was associated with decreased vaccine literacy (OR 0.80, *P* = 0.025). Factors associated with decreased vaccine hesitancy were positive beliefs about vaccines (OR 0.41, *P* < 0.001), minimal concerns over childhood vaccines (OR 0.46, *P* < 0.001), and positive interactions with HCPs (OR 0.76, *P* = 0.002). Increased hesitancy was associated with negative beliefs about vaccines (OR 3.26, *P* < 0.001) and trouble understanding information from HCPs (OR 1.44, *P* < 0.001).

**Conclusions:**

Greater vaccine literacy levels and less vaccine hesitancy correlated with positive parental beliefs about vaccines and positive interactions with HCPs. Challenges in comprehending HCP-delivered information were correlated with lower literacy and greater hesitancy.

**Supplementary Information:**

The online version contains supplementary material available at 10.1186/s12889-025-23410-y.

## Introduction

Implementation of worldwide childhood vaccination over the past several decades has resulted in a substantial decrease in cases and deaths from vaccine-preventable diseases [1]. The United States (US) Centers for Disease Control and Prevention (CDC) recommends a total of 12 vaccines for children ages 0–6 (chickenpox, coronavirus disease 2019 (COVID-19), diphtheria/tetanus/acellular pertussis (DTaP), *Haemophilus influenzae* type b (Hib), hepatitis A, hepatitis B, influenza, measles/mumps/rubella (MMR), polio, pneumococcal, and rotavirus; plus respiratory syncytial virus, depending on the mother’s vaccination status) and another two routine vaccines for children ages 7–18 (human papillomavirus and meningococcal ACWY) as well as a DTaP booster and seasonal boosters (influenza and COVID-19) [2].

In the US, early childhood vaccination rates in children born in 2019–2020 were > 90% for chickenpox, hepatitis B, MMR, and polio, but coverage rates hovered around 80% for complete DTaP (≥ 4 doses), hepatitis A (≥ 2 doses by age 35 months), Hib (primary series and booster dose), and pneumococcal (≥ 4 doses) vaccines, and were even lower for rotavirus (77%) and influenza (61%) [3]. Furthermore, a 2014 analysis of National Immunization Survey (NIS) data found that 37% of young children’s vaccination patterns did not follow the CDC-recommended schedule [4].

Parents are the natural decision-makers regarding children’s vaccinations, and various factors can influence those decisions [5]. One of these factors is vaccine literacy, which has been defined as the ability to “access, understand, and critically appraise and apply information about… vaccines… to navigate the health system, in order to make informed decisions about vaccines… and to appreciate the larger global impact of vaccines with respect to population health” [6]. Vaccine literacy is closely linked to the long-established concept of health literacy, with some overlapping and distinct components [7]. Because vaccine literacy is a relatively novel and complex concept, the literature on how parental vaccine literacy affects vaccination rates is underdeveloped, and the effect of parental vaccine literacy on pediatric vaccine uptake remains unclear [7, 8].

Another factor in parents’ decisions regarding the vaccination of their children is vaccine hesitancy, defined by a World Health Organization (WHO) working group as a “delay in acceptance or refusal of vaccination despite availability of vaccination services” [9]. Like vaccine literacy, vaccine hesitancy is a complex, context-specific, multi-factorial concept with emotional, psychological, and social determinants [9]. Hesitancy has recently been described as a motivational state rather than either a belief or a behavior [10].

Characterizing the multiple reasons for low uptake of certain vaccines and low adherence to vaccination schedules can help guide the development of strategies to improve vaccination uptake [10]. It is important to understand how vaccine literacy shapes vaccine confidence, as it lays the foundation for individuals to find points of leverage (i.e., individual knowledge, provider recommendations, community support) that help them move from hesitancy toward confidence. The goal of this study was to understand the demographic and socioeconomic characteristics associated with parents’ vaccine literacy and hesitancy and to examine the association of attitudinal, behavioral, and experiential factors with parental vaccine literacy and hesitancy. The results will inform the development of targeted and tailored vaccine interventions focused on improving scientific literacy [11].

## Methods

### Study design and respondent sample

This was a cross-sectional study consisting of an online survey of US adults conducted from January to March 2022. Survey development and recruitment have been described in a previous publication [12]. In brief, participants were recruited from the 2020–2021 National Health and Wellness Survey cohort, which is a pre-existing, general-purpose panel. We employed a quota sampling strategy to ensure that this sample was representative of the US adult population per the 2021 US Census data. A subset of adults (*N* = 692) included in this study was defined as “parents” (including those with guardian status) based on their self-report of having children aged < 18. Cognitive interviews were conducted to assess and improve the clarity and understanding of the survey questions. Feedback from these interviews was used to revise the survey for better clarity.

The study protocol was reviewed by the Pearl Institutional Review Board/Ethics Review Committee (Study Number 21-KANT-262), and the study was conducted and reported in compliance with the International Society of Pharmacoepidemiology’s Guidelines for Good Pharmacoepidemiology Practice and in accordance with the Declaration of Helsinki. We conducted informed consent processes with each participant, and approved compensation was given to those who completed the full survey.

### Outcomes of interest

For the primary analyses, parental vaccine literacy and hesitancy were defined in terms of individual survey questions (Table A1). Vaccine literacy was linked to the question, “How familiar are you with the vaccines your child should receive?” Answers of “moderately familiar” or “extremely familiar” were defined as high literacy, while answers of “somewhat familiar,” “slightly familiar,” or “not at all familiar” were defined as low literacy. Vaccine hesitancy was linked to the question, “Overall, how hesitant about childhood shots would you consider yourself to be?” Survey participants were informed that “For the purposes of this survey, the word ‘hesitant’ includes delaying the acceptance of a vaccine or vaccinations for any reason – including taking time to conduct your own research. Self-report of being “very hesitant,” “somewhat hesitant,” or “not sure” was defined as hesitancy, while answers of “not too hesitant” or “not at all hesitant” were defined as non-hesitancy.

**Fig. 1:**
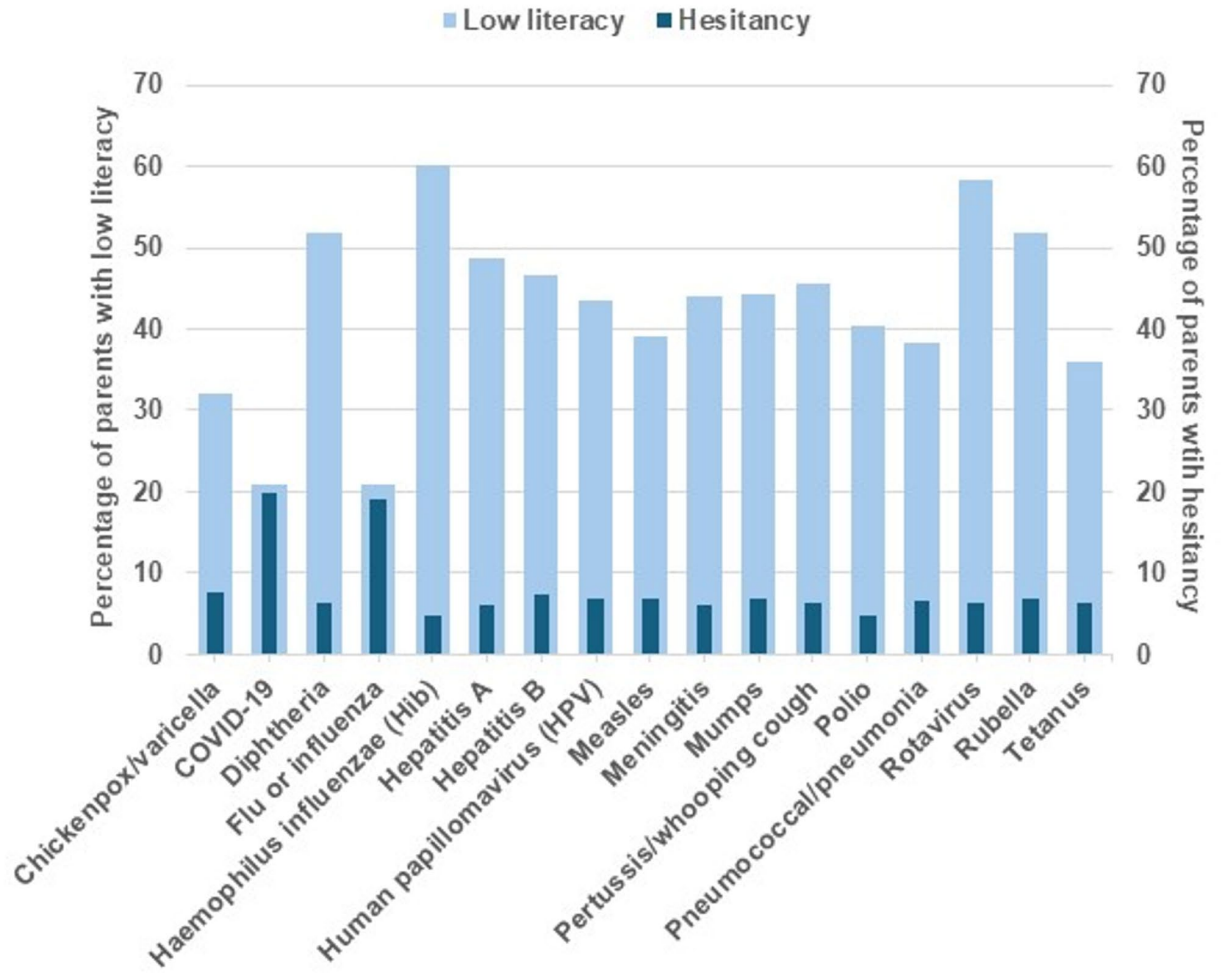
Distribution of vaccine literacy and hesitancy among parents by vaccine-preventable disease

Secondary analyses of parental literacy and hesitancy were based on additional survey questions in the context of specific vaccine-preventable diseases. Literacy (knowledge level) was evaluated with the question, “How knowledgeable are you about each of the following vaccine-preventable diseases?” (see Fig. 1 for the list of diseases queried). Answers of “I know a lot about it” or “I am somewhat knowledgeable about it” were defined as high literacy, while answers of “I have heard of it and know what it is, but not much else,” “I have heard of it but don’t know anything about it,” or “I have never heard of it before today” were defined as low literacy. Hesitancy was assessed across vaccine-preventable diseases with the question, “How would you describe your response when your child was offered each of the following vaccines?” Answers of “hesitated but accepted” or “hesitated and refused” were defined as hesitancy, and an answer of “accepted without hesitation” was defined as non-hesitancy.

### Survey questions and study variables

Survey participants were asked questions about their experiences finding and assessing information regarding pediatric vaccination and their information-seeking and decision-making practices; their interactions with healthcare providers (HCPs) about pediatric vaccines; their beliefs about the efficacy, safety, and frequency of childhood vaccines; their level of trust in scientific and medical information from various sources; the danger and severity of vaccine-preventable diseases; and the benefits and convenience of vaccination. The full survey is described in a previous publication [12]. The survey questions related to parental beliefs and self-reported behaviors are listed in the Appendix. Responses were rated on a 5-point Likert scale (e.g., 1 = “strongly disagree” or “never”; 5 = “strongly agree” or “always”) or as a binary yes/no response.

### Statistical analyses

The distribution of parental vaccine literacy and hesitancy across demographic and socioeconomic groupings (age, gender, race/ethnicity, education, income, region, and urbanicity) was assessed by bivariate analysis, with P-values determined by chi-square tests (*P* < 0.05 indicates statistical significance). Distributions are presented as numbers and percentages and were used to identify population characteristics correlated with vaccine literacy and hesitancy as defined in the primary analyses.

In the primary analyses, logistic regression models were developed to assess the associations of parental vaccine literacy and hesitancy with each survey item (i.e., each independent variable) separately, adjusting for a list of potential confounders, including age, gender, race/ethnicity, education, income, region, and urbanicity. In addition, factor analysis was conducted to identify complex interrelationships among survey questions and group items into integrated concepts. Correlations were tested between each pair of survey questions, and no extremely correlated pairs (absolute value of correlation coefficient > 0.8) were found. The number of factors was determined based on parallel analysis, and variables with an absolute value of factor loading > 0.3 were considered significant contributors to a factor. The identified factors were assessed for association with vaccine literacy and hesitancy by regression models, with adjustment for the same covariates. Results of the regression analyses are presented as odds ratios (ORs) and 95% confidence intervals (CIs), with P-values < 0.05 indicating statistical significance.

In the secondary analyses, parental vaccine literacy and hesitancy were measured in relation to various vaccine-preventable diseases (see Fig. 1). The definitions described above were used to categorize parents as having high literacy or being hesitant. Finally, correlations of literacy about and hesitancy toward specific pairs of vaccines were assessed by pairwise association analysis using chi-square tests. P-values < 0.05 indicated that literacy about or hesitancy toward the first vaccine in a pair was correlated with literacy about or hesitancy toward the second vaccine and were considered statistically significant.

## Results

### Characteristics of the study population

In this survey sample of 692 parents of children aged < 18, most participants had high vaccine literacy (74.4%), whereas vaccine hesitancy was evenly distributed (49.7% hesitant, 50.3% non-hesitant; Table 1). The majority of participants were non-Hispanic Whites (66.3%), females (56.9%), and aged 36–45 (51.2%). A plurality of participants was college- (33.4%) or graduate school-educated (30.3%), had a household income ≥$100,000 (42.7%), and lived in an urban setting (49.0%).

**Table 1:** Distribution of vaccine literacy and hesitancy among parents by demographic and socioeconomic characteristics

	Total *N* (%)	Vaccine literacy				Vaccine hesitancy		
		High literacy *n* (%)	Low literacy *n* (%)	*P*-value^A^		Hesitant *n* (%)	Non-hesitant *n* (%)	*P*-value^A^
Overall	692 (100)	515 (74.4)	177 (25.6)			344 (49.7)	348 (50.3)	
Age				0.786				< 0.001
18–26	29 (4.2)	20 (3.9)	9 (5.1)			19 (5.5)	10 (2.9)	
27–35	188 (27.2)	143 (27.8)	45 (25.4)			117 (34.0)	71 (20.4)	
36–45	354 (51.2)	260 (50.5)	94 (53.1)			172 (50.0)	182 (52.3)	
>46	121 (17.5)	92 (17.9)	29 (16.4)			36 (10.5)	85 (24.4)	
Gender				0.039				0.040
Female	394 (56.9)	281 (54.6)	113 (63.8)			182 (52.9)	212 (60.9)	
Male	298 (43.1)	234 (45.4)	64 (36.2)			162 (47.1)	136 (39.1)	
Race/ethnicity				0.021				0.004
Non-Hispanic White	459 (66.3)	355 (68.9)	104 (58.8)			233 (67.7)	226 (64.9)	
Non-Hispanic Black	69 (10.0)	47 (9.1)	22 (12.4)			36 (10.5)	33 (9.5)	
Asian	50 (7.2)	29 (5.6)	21 (11.9)			15 (4.4)	35 (10.1)	
Hispanic	92 (13.3)	66 (12.8)	26 (14.7)			54 (15.7)	38 (10.9)	
Others	22 (3.2)	18 (3.5)	4 (2.3)			6 (1.7)	16 (4.6)	
Education				0.011				0.035
High school or less	100 (14.5)	64 (12.4)	36 (20.3)			55 (16.0)	45 (12.9)	
Some college	151 (21.8)	106 (20.6)	45 (25.4)			70 (20.3)	81 (23.3)	
College	231 (33.4)	177 (34.4)	54 (30.5)			101 (29.4)	130 (37.4)	
Graduate school	210 (30.3)	168 (32.6)	42 (23.7)			118 (34.3)	92 (26.4)	
Income				0.006				0.950
<$50,000	147 (21.6)	94 (18.6)	53 (29.9)			73 (21.4)	74 (21.7)	
$50,000 - $100,000	244 (35.8)	190 (37.6)	54 (30.5)			124 (36.4)	120 (35.2)	
≥$100,000	291 (42.7)	221 (43.8)	70 (39.5)			144 (42.2)	147 (43.1)	
Region				0.510				0.497
Northeast	131 (19.2)	99 (19.5)	32 (18.4)			59 (17.5)	72 (21.0)	
Midwest	160 (23.5)	112 (22.1)	48 (27.6)			77 (22.8)	83 (24.2)	
South	251 (36.9)	192 (37.9)	59 (33.9)			133 (39.3)	118 (34.4)	
West	139 (20.4)	104 (20.5)	35 (20.1)			69 (20.4)	70 (20.4)	
Urbanicity				0.002				< 0.001
Urban	339 (49.0)	272 (52.8)	67 (37.9)			211 (61.3)	128 (36.8)	
Suburban	264 (38.2)	180 (35.0)	84 (47.5)			98 (28.5)	166 (47.7)	
Rural	89 (12.9)	63 (12.2)	26 (14.7)			35 (10.2)	54 (15.5)	

^A^ P-values indicate the statistical significance of chi-square tests comparing the distributions of hesitant vs. non-hesitant parents and parents with high vs. low literacy across the different categories

### Demographic and socioeconomic characteristics associated with vaccine literacy and hesitancy

Vaccine literacy differed significantly by gender, race/ethnicity, education, income, and urban residence (Table 1). Compared to parents with self-reported high vaccine literacy, those who reported low vaccine literacy were more likely to be mothers (63.8% mothers among those with low literacy vs. 54.6% mothers among those with high literacy). Parents who reported low vaccine literacy were more likely to have lower education (20.3% of those with low literacy had a high school diploma or less vs. 12.4% of those with high literacy), lower income (29.9% of those with low literacy made <$50,000/year vs. 18.6% of those with high literacy), and live in suburban residential settings (47.5% of those with low literacy lived in suburbs vs. 35.0% of those with high literacy). In addition, high vaccine literacy was observed to be more frequent among non-Hispanic White parents (68.9%) but less frequent among non-Hispanic Black (9.1%), Asian (5.6%), and Hispanic parents (12.8%).

Vaccine hesitancy differed significantly by age, gender, race/ethnicity, education, and urban residency area (Table 1). Compared to those who reported non-hesitancy toward children’s vaccines, parents who were vaccine hesitant were more likely to be younger (e.g., 34.0% of hesitant parents were aged 27–35 vs. 20.4% of those who were non-hesitant), and male (47.1% of hesitant parents were male vs. 39.1% of non-hesitant parents). Vaccine hesitancy was observed to be more frequent among non-Hispanic White (67.7% of hesitant vs. 64.9% of non-hesitant) and Hispanic (15.7% of hesitant vs. 10.9% of non-hesitant) parents but less frequent in Asian (4.4% of hesitant vs. 10.1% of non-hesitant) parents. Those who reported vaccine hesitancy were more likely to have higher education level (34.3% of hesitant parents attended graduate school vs. 26.4% of non-hesitant parents) and live in an urban setting (61.3% of hesitant parents had urban residence vs. 36.8% of non-hesitant parents).

### Association of attitudes, behaviors, beliefs, and experiences with vaccine literacy and hesitancy

Table 2 shows the association of individual survey items with vaccine literacy and hesitancy (primary analyses; unadjusted results are given in Supplementary Table S1). High vaccine literacy was associated with a decrease in vaccine hesitancy (OR 0.76, 95% CI 0.63–0.91, *P* = 0.004), whereas vaccine hesitancy was not significantly associated with high vaccine literacy (OR 1.07, 95% CI 0.92–1.23, *P* = 0.371).

**Table 2:** Associations of behaviors, beliefs, and experiences with parental vaccine literacy and hesitancy^A^

	Vaccine literacy (high vs. low)			Vaccine hesitancy (yes vs. no)	
	OR (95% CI)	P-value		OR (95% CI)	P-value
**Vaccine hesitancy**					
Overall, how hesitant about childhood vaccines would you consider yourself to be? (1–5, very hesitant - not at all)	1.07 (0.92, 1.23)	0.371		-	
If you had another infant today, would you want him/her to get all the recommended shots? (yes/no)	1.82 (1.09, 3.02)	**0.021**		-	
**Vaccine literacy (1–5**,** not at all - extremely familiar)**					
Familiarity with vaccines your child should receive	-			0.76 (0.63, 0.91)	**0.004**
Familiarity with the schedule on your child’s vaccines	-			0.85 (0.71, 1.02)	0.083
**When reading information regarding vaccines provided by a health care professional**,** do you… (1–5**,** never - always)**					
Find the print too small to read	0.94 (0.82, 1.09)	0.414		1.57 (1.38, 1.81)	**< 0.001**
Find characters and words that you do not understand	0.96 (0.83, 1.11)	0.576		1.70 (1.48, 1.97)	**< 0.001**
Find the text too hard to understand	0.85 (0.73, 0.98)	**0.026**		1.76 (1.53, 2.04)	**< 0.001**
Need a long time to read and understand the text	0.99 (0.85, 1.14)	0.842		1.63 (1.42, 1.88)	**< 0.001**
Need someone to help you read the information	0.97 (0.83, 1.13)	0.678		1.96 (1.68, 2.30)	**< 0.001**
**Since your child was born**,** you have… (1–5**,** never - always)**					
Collected information on vaccinations from more than one source	1.68 (1.41, 1.99)	**< 0.001**		1.35 (1.15, 1.58)	**< 0.001**
Looked for information on the vaccines you were interested in	1.68 (1.43, 1.99)	**< 0.001**		1.30 (1.12, 1.52)	**0.001**
Understood the obtained information (on vaccines)	2.02 (1.67, 2.47)	**< 0.001**		0.87 (0.73, 1.03)	0.110
Discussed your thoughts about your child’s vaccinations with medical staff	1.69 (1.44, 1.99)	**< 0.001**		1.00 (0.87, 1.16)	0.975
Applied the obtained information to make decisions regarding your child’s vaccinations	1.69 (1.43, 2.02)	**< 0.001**		0.87 (0.74, 1.01)	0.076
**When your child needed or was recommended a vaccine**,** you have… (1–5**,** never - always)**					
Considered the credibility of the information about the vaccines	1.66 (1.41, 1.97)	**< 0.001**		1.16 (0.99, 1.35)	0.064
Checked whether the information about the vaccines was valid and reliable	1.71 (1.46, 2.01)	**< 0.001**		1.25 (1.08, 1.46)	**0.003**
Looked for information that helped you make health-related decisions for your child	1.78 (1.50, 2.11)	**< 0.001**		1.19 (1.02, 1.40)	**0.024**
**When/after talking to a healthcare professional about vaccines for your child… (1–5**,** never - always)**					
How often do you feel like you are able to give them all the information they need to help you	2.49 (1.99, 3.14)	**< 0.001**		0.60 (0.49, 0.73)	**< 0.001**
How often do you feel like you are able to ask all the questions you have	2.13 (1.73, 2.64)	**< 0.001**		0.75 (0.62, 0.91)	**0.003**
How often do you feel like you are able to make sure they explain anything that you do not understand	2.18 (1.78, 2.69)	**< 0.001**		0.74 (0.61, 0.89)	**0.001**
How likely are you to do your own background reading or gather additional information	1.65 (1.41, 1.95)	**< 0.001**		1.64 (1.40, 1.93)	**< 0.001**
How well do you think he or she listens to your questions and concerns about your child’s routine vaccinations	2.74 (2.17, 3.49)	**< 0.001**		0.92 (0.76, 1.12)	0.401
How often do they answer in a way that is easy for you to understand	1.64 (1.39, 1.93)	**< 0.001**		0.75 (0.64, 0.87)	**< 0.001**
How much influence do you feel you have on your child’s vaccination schedule	2.27 (1.90, 2.73)	**< 0.001**		0.87 (0.75, 1.01)	0.072
**How much do you agree with each of the following statements? (1–5**,** strongly disagree - strongly agree)**					
Healthcare professionals give out too many vaccines	0.99 (0.86, 1.13)	0.864		2.29 (1.97, 2.69)	**< 0.001**
Vaccines are a good way to protect my family/friends	1.46 (1.20, 1.79)	**< 0.001**		0.28 (0.22, 0.36)	**< 0.001**
I do not like the idea of vaccines for my child	0.85 (0.74, 0.98)	**0.024**		2.47 (2.12, 2.90)	**< 0.001**
Vaccines are generally safe	1.59 (1.29, 1.97)	**< 0.001**		0.36 (0.28, 0.45)	**< 0.001**
Vaccines can cause immediate, short-term side effects (such as fever, pain, etc.)	1.22 (1.02, 1.45)	**0.032**		0.98 (0.83, 1.16)	0.814
Vaccines are a way to take good care of my child now and in the future	1.51 (1.24, 1.83)	**< 0.001**		0.38 (0.30, 0.48)	**< 0.001**
My child is not afraid of shots/needles	1.26 (1.09, 1.46)	**0.002**		1.07 (0.93, 1.22)	0.346
Vaccines are effective	1.63 (1.32, 2.01)	**< 0.001**		0.29 (0.22, 0.37)	**< 0.001**
Vaccines contain dangerous ingredients	0.90 (0.78, 1.04)	0.170		2.38 (2.03, 2.81)	**< 0.001**
Vaccines can cause conditions such as autism or infertility	0.88 (0.77, 1.02)	0.083		2.43 (2.09, 2.86)	**< 0.001**
There is no need for my child to get vaccinated because everybody else does	0.93 (0.81, 1.07)	0.292		2.42 (2.08, 2.83)	**< 0.001**
Vaccines are important as they are beneficial to the community	1.47 (1.21, 1.78)	**< 0.001**		0.35 (0.27, 0.43)	**< 0.001**
I follow advice from friends/family/colleagues who think it is important to get vaccinated	1.24 (1.05, 1.46)	**0.010**		1.09 (0.94, 1.28)	0.246
I follow advice from friends/family/colleagues who think vaccination is NOT important, safe, or effective	0.99 (0.86, 1.14)	0.931		2.13 (1.84, 2.48)	**< 0.001**
I would prefer my child gain protection from an illness by catching the illness themselves rather than getting the vaccine	0.97 (0.85, 1.12)	0.715		2.24 (1.93, 2.61)	**< 0.001**
Vaccines can cause long-term side effects	0.90 (0.77, 1.05)	0.173		2.32 (1.96, 2.76)	**< 0.001**
Healthy children do not need vaccinations	0.87 (0.76, 1.00)	0.055		2.44 (2.09, 2.87)	**< 0.001**
I am more likely to trust vaccines that have been around longer, compared to newer vaccines	1.48 (1.21, 1.81)	**< 0.001**		0.80 (0.66, 0.96)	**0.019**
I trust science to develop safe and effective vaccines	1.49 (1.22, 1.81)	**< 0.001**		0.37 (0.29, 0.47)	**< 0.001**
I trust the government to ensure vaccines are safe and effective	1.28 (1.09, 1.50)	**0.002**		0.67 (0.57, 0.78)	**< 0.001**
Vaccination should be required for children to attend school	1.33 (1.14, 1.56)	**< 0.001**		0.62 (0.52, 0.72)	**< 0.001**
It is okay for the government to mandate vaccination	1.21 (1.05, 1.39)	**0.008**		0.76 (0.66, 0.87)	**< 0.001**
People should have the right to be medically exempt from receiving vaccines	1.16 (0.99, 1.36)	0.062		1.60 (1.37, 1.88)	**< 0.001**
People should have the right to claim religious exemptions from receiving vaccines	1.06 (0.92, 1.22)	0.403		1.89 (1.63, 2.20)	**< 0.001**
It is acceptable for companies/employers to require vaccines for employees to attend/return to work	1.23 (1.07, 1.43)	**0.004**		0.71 (0.61, 0.82)	**< 0.001**
People that are allergic to ingredients in the vaccine should have the right to be exempt from receiving vaccines	1.39 (1.14, 1.69)	**0.001**		0.88 (0.74, 1.06)	0.187
Children get more shots than are good for them	0.99 (0.85, 1.14)	0.840		2.60 (2.20, 3.11)	**< 0.001**
I believe that many of the illnesses that shots prevent are severe	1.12 (0.94, 1.34)	0.196		0.61 (0.50, 0.72)	**< 0.001**
It is better for my child to develop immunity by getting sick than to get a shot	0.93 (0.81, 1.07)	0.305		2.49 (2.14, 2.94)	**< 0.001**
It is better for children to get fewer vaccines at the same time	1.11 (0.94, 1.32)	0.227		1.89 (1.58, 2.27)	**< 0.001**
I trust the information I receive about shots	1.30 (1.10, 1.54)	**0.003**		0.41 (0.33, 0.51)	**< 0.001**
I am able to openly discuss my concerns about shots with my child’s healthcare professional	1.84 (1.51, 2.26)	**< 0.001**		0.50 (0.41, 0.62)	**< 0.001**
How much do you trust your child’s doctor (1–10)	1.40 (1.24, 1.59)	**< 0.001**		0.64 (0.55, 0.73)	**< 0.001**
Children start receiving vaccines when they are too young (yes/no)	1.12 (0.74, 1.69)	0.607		4.90 (3.34, 7.26)	**< 0.001**
**How concerned are you about each of the following items? (1–5**,** very concerned- not at all concerned)**					
Your child might have a serious side effect from a shot	0.85 (0.73, 1.00)	**0.043**		0.66 (0.57, 0.76)	**< 0.001**
Any one of the childhood shots might not be safe	0.93 (0.80, 1.07)	0.317		0.43 (0.36, 0.50)	**< 0.001**
A shot might not prevent disease	0.88 (0.75, 1.02)	0.093		0.43 (0.37, 0.51)	**< 0.001**
**Who benefits the MOST when you/children receive all of the recommended vaccines? (yes/no)**					
The child/myself	0.93 (0.63, 1.36)	0.699		0.26 (0.18, 0.37)	**< 0.001**
The community	1.50 (1.03, 2.20)	**0.038**		0.29 (0.20, 0.41)	**< 0.001**
The healthcare provider	0.72 (0.48, 1.09)	0.117		2.11 (1.43, 3.14)	**< 0.001**
The government	1.58 (0.89, 2.96)	0.132		2.91 (1.71, 5.12)	**< 0.001**
The vaccine/pharmaceutical companies	1.49 (0.97, 2.32)	0.073		2.45 (1.66, 3.65)	**< 0.001**
**How convenient is it for you to get to the following? (1–5**,** not at all convenient - extremely convenient)**					
Routine doctor’s visits (yearly visit, prescription renewal/ refills)	1.67 (1.37, 2.05)	**< 0.001**		0.82 (0.67, 0.99)	**0.041**
Non-routine doctor’s visits (sick or health concern visits)	1.45 (1.22, 1.74)	**< 0.001**		0.92 (0.78, 1.09)	0.353
Pharmacy	1.80 (1.46, 2.23)	**< 0.001**		0.65 (0.53, 0.79)	**< 0.001**
Specialists (not general or family practitioner)	1.36 (1.13, 1.63)	**0.001**		1.05 (0.89, 1.24)	0.588
Nearest hospital	1.44 (1.18, 1.77)	**< 0.001**		0.79 (0.65, 0.95)	**0.012**
Routine dental visits (i.e., cleanings)	1.55 (1.26, 1.90)	**< 0.001**		0.79 (0.65, 0.95)	**0.014**
Non-routine dental visits (i.e., toothache, fillings, root canals, other procedures)	1.39 (1.16, 1.67)	**< 0.001**		1.03 (0.87, 1.21)	0.761

CI, confidence interval; OR, odds ratio

^A^All OR values were adjusted for age, gender, race/ethnicity, education, income, region, and urbanicity. Unadjusted data are shown in Supplementary Table S1. P-values in bold indicate statistical significance

As overall themes, the following behaviors, beliefs, and experiences were associated with increased vaccine literacy: researching, understanding, discussing, evaluating, and applying information about vaccines; positive interactions with HCPs about pediatric vaccination; belief in the safety and efficacy of vaccines; trust in the government, science, HCPs, and advice from family and friends; and self-reported convenience in accessing doctors’ and dentists’ offices, hospitals, and pharmacies (Table 2). The strongest correlates of high vaccine literacy were all found in the relationship with the HCP: the belief that the HCP listens to questions and concerns (OR 2.74, 95% CI 2.17–3.49, *P* < 0.001), the feeling of being able to give them all the information they need to help you (OR 2.49, 95% CI 1.99–3.14, *P* < 0.001), and the feeling of having a lot of influence over the pediatric vaccination schedule (OR 2.27, 95% CI 1.90–2.73, *P* < 0.001). Items that were most strongly associated with reduced literacy were finding information on vaccines too hard to understand (OR 0.85, 95% CI 0.73–0.98, *P* = 0.026), a general dislike of the idea of childhood vaccines (OR 0.85, 95% CI 0.74–0.98, *P* = 0.024), and concern about serious side effects from a shot (OR 0.85, 95% CI 0.73-1.00, *P* = 0.043).

The following behaviors, beliefs, and experiences were generally associated with increased vaccine hesitancy: difficulty reading and understanding vaccine information, regularly researching and considering the validity/credibility of vaccine information, and the belief that vaccines are unnecessary or potentially unsafe (Table 2). The correlates of vaccine hesitancy were the beliefs that children start receiving vaccines when they are too young (OR 4.90, 95% CI 3.34–7.26, *P* < 0.001), that the government benefits the most when children receive all of the recommended vaccines (OR 2.91, 95% CI 1.71–5.12, *P* < 0.001), that children get more shots than is good for them (OR 2.60, 95% CI 2.20–3.11, *P* < 0.001), and that it is better for a child to develop immunity by getting sick than to get a shot (OR 2.49, 95% CI 2.14–2.94, *P* < 0.001). Conversely, items that were most strongly associated with decreased hesitancy were the beliefs that the child (OR 0.26, 95% CI 0.18–0.37, *P* < 0.001) or the community (OR 0.29, 95% CI 0.20–0.41, *P* < 0.001) benefits the most when children receive all of the recommended vaccines, that vaccines are a good way to protect family/friends (OR 0.28, 95% CI 0.22–0.36, *P* < 0.001), and that vaccines are effective (OR 0.29, 95% CI 0.22–0.37, *P* < 0.001).

### Factors associated with vaccine literacy and hesitancy

We conducted an exploratory factor analysis to further investigate relationships in the data and ascribe the set of survey questions to specific domains comprised of a smaller number of items. All survey questions shown in Table 2 were examined except for those constructed as binary yes/no questions (e.g., the questions about who benefits most) and the questions about convenience. Supplementary Table S2 shows the derivation of 7 factors from the survey questions, categorized as follows: (1) negative beliefs about vaccines, (2) positive beliefs about vaccines, (3) active information-seeking behavior, (4) trouble understanding information from HCPs, (5) positive interactions with HCPs, (6) beliefs about vaccination requirements, and (7) concerns about children’s vaccines. Note that for factor 7, concern was rated in reverse compared to the other questions due to the foundational question’s wording (highest concern was the lowest number) on the Likert scale.

The association of these 7 factors with vaccine literacy and hesitancy is shown in Table 3 (unadjusted results are shown in Supplementary Table S3). Positive interaction with HCPs was the strongest correlate of high vaccine literacy (OR 2.24, 95% CI 1.83–2.77, *P* < 0.001), followed by active information-seeking behavior (OR 1.88, 95% CI 1.55–2.30, *P* < 0.001), positive beliefs about vaccines (OR 1.39, 95% CI 1.15–1.70, *P* = 0.001), and beliefs about vaccination requirements (OR 1.21, 95% CI 1.01–1.46, *P* = 0.041). Conversely, trouble understanding information from HCPs (OR 0.80, 95% CI 0.66–0.97, *P* = 0.025) and minimal concerns about children’s vaccines (OR 0.83, 95% CI 0.69-1.00, *P* = 0.049) were correlated with reduced vaccine literacy. Two factors were correlated with vaccine hesitancy: negative beliefs about vaccines (OR 3.26, 95% CI 2.62–4.12, *P* < 0.001) and trouble understanding information from HCPs (OR 1.44, 95% CI 1.21–1.71, *P* < 0.001). Three factors were correlated with reduced hesitancy: positive beliefs about vaccines (OR 0.41, 95% CI 0.33–0.50, *P* < 0.001), minimal concerns about children’s vaccines (OR 0.46, 95% CI 0.38–0.56, *P* < 0.001), and positive interactions with HCPs (OR 0.76, 95% CI 0.64–0.91, *P* = 0.002).

**Table 3:** Association of factors with vaccine literacy and hesitancy^A^

	Vaccine literacy (high vs. low)			Vaccine hesitancy (yes vs. no)	
Factor	OR (95% CI)	P-value		OR (95% CI)	P-value
1: Negative beliefs about vaccines	0.89 (0.73, 1.08)	0.239		3.26 (2.62, 4.12)	**< 0.001**
2: Positive beliefs about vaccines	1.39 (1.15, 1.70)	**0.001**		0.41 (0.33, 0.50)	**< 0.001**
3: Active information-seeking behavior	1.88 (1.55, 2.30)	**< 0.001**		1.18 (0.99, 1.41)	0.064
4: Trouble understanding information from HCPs	0.80 (0.66, 0.97)	**0.025**		1.44 (1.21, 1.71)	**< 0.001**
5: Positive interactions with HCPs	2.24 (1.83, 2.77)	**< 0.001**		0.76 (0.64, 0.91)	**0.002**
6: Beliefs about vaccination requirements	1.21 (1.01, 1.46)	**0.041**		0.85 (0.72, 1.01)	0.059
7: Concerns about children’s vaccines	0.83 (0.69, 1.00)	**0.049**		0.46 (0.38, 0.56)	**< 0.001**

CI, confidence interval; HCP, healthcare provider; OR, odds ratio

^A^All OR values were adjusted for age, gender, race/ethnicity, education, income, region, and urbanicity. Unadjusted data are shown in Supplementary Table S3. *P*-values in bold indicate statistical significance

### Literacy about and hesitancy toward specific vaccines

Finally, we assessed vaccine literacy and hesitancy in the context of specific vaccine-preventable diseases. Figure 1 shows the percentage of parents with low literacy about and hesitancy toward specific vaccines. Overall, low literacy (range 21.0–60.1%) was more common than hesitancy (range 4.8–19.8%) for the specific vaccines. Low vaccine literacy was most frequently observed for Hib (60.1% of parents) and rotavirus (58.2%), followed by rubella (51.9%) and diphtheria (51.7%). Low literacy was least common for COVID-19 and influenza (both 21.0%), the same diseases for which vaccine hesitancy was most common (19.8% and 19.1%, respectively). Supplementary Tables S4 and S5 show the pairwise association of literacy about and hesitancy toward vaccination for each vaccine-preventable disease. These tests indicated that high literacy about several specific vaccines (e.g., chickenpox, COVID-19, diphtheria, influenza, Hib, pneumococcal, rotavirus, rubella, and tetanus) correlated with high literacy about other vaccines (Supplementary Table S4). In contrast, hesitancy was clustered around COVID-19 and influenza. Hesitancy regarding these two vaccines correlated with hesitancy toward other vaccines, but no such trend was observed for the other vaccines (Supplementary Table S5).

The graph shows the percentage of parents with low vaccine literacy (left axis) and vaccine hesitancy (right axis), regarding each of the indicated vaccine-preventable diseases. Pairwise correlations of literacy and hesitancy for specific diseases are shown in Supplementary Tables S4-S5.

## Discussion

This study identified the demographic and socioeconomic characteristics associated with US parents’ vaccine literacy and hesitancy as well as behaviors, beliefs, and experiences that were associated with vaccine literacy and hesitancy. Three out of four parents were defined as having high literacy, while half of parents were self-described as hesitant. Factor analysis revealed several consistent patterns in the data: positive beliefs about vaccines and positive interactions with HCPs correlated with greater literacy and less hesitancy, while trouble understanding information from HCPs had the opposite effects. Our data also showed that literacy about and hesitancy toward COVID-19 and influenza vaccines were consistently correlated with literacy and hesitancy regarding other vaccines.

In our study, high literacy was associated with non-hesitancy, which is consistent with previous work that has characterized vaccine literacy as being situated at the intersection of individuals’ sociodemographic characteristics and psychological attitudes [13]. Our survey identified parental characteristics that are associated with low vaccine literacy; these included male sex, non-White race, low education and income levels, and suburban residence. These results are consistent with previous studies of vaccine literacy in adults, which showed that vaccine literacy differed by age, sex, education level, and socioeconomic status [14–17]. However, these previous studies captured the vaccine literacy of adults regarding vaccination for themselves, not their children, so the current study provides valuable novel information on the parental perspective. Parents with low literacy characteristics might benefit from more and better information from HCPs regarding childhood vaccination, especially given our finding that high literacy was strongly associated with a positive relationship with the HCP. However, the ability to develop such relationships with HCPs may also be shaped by structural barriers in the American healthcare system—including cost, time, and access—particularly for those with a lower socioeconomic status [18], highlighting an important area for future research.

Trouble understanding information given by HCPs (a multivariable factor) and finding information on vaccines too hard to understand (an individual survey item) were both associated with reduced literacy in the current study. Challenges with information provided by HCPs could prompt parents to seek alternative sources of information on vaccines, potentially exposing them to erroneous information. In a recent online survey of Italian parents of school-aged children (*N* = 2,301), 27.7% of respondents reported getting vaccine information from non-institutional sources (TV/newspaper, internet, or friends/family) [19]. Characteristics associated with the use of non-institutional information sources included vaccine hesitancy (OR 2.52), perceived low quality of the healthcare system (OR 2.51), having older children (OR 1.60–1.69 for middle and high school vs. elementary), being male (OR 1.42), and a personal desire for more information (OR 1.44).

Previous reviews of vaccine literacy have found mixed effects of parental literacy on childhood vaccination in countries as diverse as China, India, Israel, the Netherlands, and the US, both in a vaccine-specific context and with general routine vaccination series [7, 8]. This highlights the complex, multi-faceted nature of this outcome measure. Vaccine literacy has been shown to be more strongly associated with vaccine intent and acceptance than with actual vaccine uptake [20, 21]. Some reasons for this may be related to the fact that vaccines protect against a disease that has never been contracted, that vaccines involve injection of a foreign substance into the body, and that vaccines are perceived to contribute more to community health than personal health [21], and these factors speak to the perceptions of importance/imminence, safety, and benefit that parents have about childhood vaccinations. The gap between vaccine intent and uptake may also be influenced by factors such as access to care, logistical barriers (e.g., inconvenient clinic times or locations), and other contextual factors [22]. This may explain in part why some studies have found that higher parental vaccine literacy was unassociated with child vaccination status or even negatively associated with willingness to vaccinate in various contexts [23–25]. The current study provides novel insight into this complex body of evidence by starting one step earlier and investigating the determinants of parental vaccine literacy itself.

Our study population was evenly divided between hesitant and non-hesitant parents. This contrasts with other surveys that found hesitancy rates under 10% (6.9% in the Italian survey described above [19] and 6.1% in another survey of US parents [26]), likely due to the fact that our definition of hesitancy included not only complete refusal but also delay, as well as a “not sure” category, which encompassed a broader range of the hesitancy spectrum than the validated scales of measurement used in the other surveys. Another survey of US parents that quantified vaccine hesitancy as low, medium, or high based on the Parent Attitudes about Childhood Vaccines short scale found that 28% of respondents were in the combined medium/high hesitancy group [27]. However, a difference in parents’ self-perception of hesitancy vs. quantitative assessments of hesitancy may arise due to our use of a definition that could conflate vaccine hesitancy with vaccination behavior (i.e., a lack of vaccine acceptance).

A decade of NIS data (2011–2021) in the US indicates that vaccine uptake is increasing overall for the core 7-vaccine series consisting of ≥ 4 doses of DTaP, ≥ 3 doses of polio, ≥ 1 dose of measles-containing vaccine, ≥ 3 or ≥ 4 doses of Hib, ≥ 3 doses of hepatitis B, ≥ 1 dose of chickenpox, and ≥ 4 doses of pneumococcal conjugate vaccine [28] which could be impacted by changing levels of hesitancy among changes in other behavioral, logistical, and contextual factors [22]. The percentage of children receiving this combination of vaccinations with no delays by age 24 months increased from 21.1% in 2011 to 35.9% in 2021. Furthermore, the NIS showed stable rates of childhood vaccinations at the national level early in the COVID-19 pandemic (2020-21), except for the 7-vaccine series in children living below the poverty line or in rural areas [29]. While there was a small increase in hesitancy during the pandemic, it returned to baseline by July 2022 [30]. Nevertheless, hesitancy still exists, as suggested by suboptimal vaccine uptake for certain vaccines in certain populations [3]. This is illustrated in a comprehensive assessment of US vaccination claims between January 2020 and August 2022 compared to 2018–2019, which showed that post-pandemic vaccination rates in children aged 0–2 had returned to nearly baseline levels for all vaccines except MMR, but those in children aged 4–6 had not [31].

In the current study, parental beliefs about vaccines were the factors with the largest effect on hesitancy, with positive beliefs decreasing hesitancy and negative beliefs increasing it. Specific beliefs that were most strongly associated with vaccine hesitancy/non-hesitancy among parents focused on child safety (i.e., age at vaccination and concerns about side effects), child benefit (vs. the government or community), the value of vaccines vs. natural immunity, and vaccine efficacy. These beliefs mirror those reported as reasons for parental hesitancy in several studies of specific childhood vaccines whose coverage rates are lower than recommended: MMR [32], rotavirus [33], and influenza [34]. Such beliefs are incorporated into the WHO’s Vaccine Hesitancy Scale in items addressing the benefits, safety, and efficacy of vaccines [35]. While positive interactions with HCPs decreased parental vaccine hesitancy in the factor analysis, the logistic regression analyses showed that other relationships (friends/family/colleagues who think vaccination is/is not important) can also have an impact on parents’ vaccination decisions (see Table 2). Given the heterogeneity of the beliefs and social norms in the various subpopulations, it is likely that a broader approach, beyond strengthening the HCP relationship, will be required to improve parental vaccine acceptance.

Since literacy and hesitancy may differ across different vaccines, we assessed these outcomes in the context of different vaccine-preventable childhood diseases. Literacy and hesitancy rates were quite different when defined overall by a single survey question (25.6% low literacy, 49.7% hesitant) than when assessed for individual vaccines (range 21.0-60.1% for low literacy, 4.8-19.8% for hesitancy). Interestingly, hesitancy was highest for the two vaccines with the lowest rate of low literacy, COVID-19 and influenza (see Fig. 1). This finding is consistent with studies that found high literacy to be associated with lower vaccine uptake [23–25], but since it was only true for two vaccines, it also shows that hesitancy can be vaccine-specific and should be monitored to detect changes that may impact uptake and coverage rates among specific populations and in relation to vaccine type [36]. Both COVID-19 and influenza are diseases that are sometimes perceived as mild [37–41], with vaccines whose effectiveness varies by season [42, 43], and these factors, along with the annual updates of vaccine composition, may affect parents’ belief in their necessity.

The primary limitation of this study was that the definitions of literacy and hesitancy were based on individual survey items rather than a multi-item standardized scale, limiting the direct comparability of our findings to other studies using such scales. Literacy was variously defined as familiarity with vaccines (primary analyses) and knowledge about vaccine-preventable diseases (secondary analyses), neither of which captures the full scope of finding, understanding, and judging immunization-related information in more formal definitions found in the literature [6, 8, 14, 21]. Furthermore, knowledge and familiarity are vaccine- and life stage-specific. Thus, these variations may limit the interpretive and comparative value of the results. The survey did provide a standard definition of hesitancy (see Methods section) to the participants before they answered questions about hesitancy, and the survey questions were modeled on published methods of defining vaccine literacy [23, 44, 45] and vaccine hesitancy [46, 47]. However, we do not know the reasons for hesitancy as defined for participants during the survey. A recent review of 14 published measures of vaccine confidence reclassified vaccine hesitancy, describing it as a motivational state that is distinct from and intermediate between beliefs and behaviors [10]. In an environment of evolving definitions and constructs in the field of vaccine confidence, and within the limitations of a cross-sectional survey, we chose to rely on simple self-reported measures for the purposes of the current analysis, noting that they are subject to perception bias. Finally, since the goal of this study was to understand the factors contributing to parental vaccine hesitancy and literacy, the self-reported behaviors assessed here were limited to information-seeking, discussions with healthcare providers, and applying information to vaccination decisions; they did not include actual vaccination uptake.

In conclusion, this study identified sociodemographic characteristics as well as attitudinal, behavioral, and experiential factors associated with vaccine literacy and vaccine hesitancy in a sample of US parents. The findings highlight positive beliefs about vaccines, positive interactions with HCPs, and provision of clear and simple information as avenues for increasing vaccine literacy and decreasing vaccine hesitancy in this population.

## Electronic supplementary material

Below is the link to the electronic supplementary material.

Supplementary Material 1

## Data Availability

All data analyzed during this study are included in this published article and its supplementary information files.

## Abbreviations

CDC: Centers for Disease Control and Prevention
CI: Confidence interval
COVID-19: Coronavirus disease 2019
DTaP: Diphtheria/tetanus/acellular pertussis
HCP: Healthcare provider
Hib: Haemophilus influenzae type b
MMR: Measles/mumps/rubella
NIS: National immunization survey
OR: Odds ratio
US: United States
WHO: World Health Organization
