# Supplementary material for: Factors associated with parental literacy and hesitancy toward pediatric vaccination
Source: BMC Public Health. 2025 Jul 2;25:2201. doi: 10.1186/s12889-025-23410-y (PMC12219603; doi:10.1186/s12889-025-23410-y)
Supplement: Supplementary file 1 — Supplementary Material 1 [file 12889_2025_23410_MOESM1_ESM.docx]

## Appendix

### Table A1. Survey questions related to parental beliefs and behaviors

| **Numbers** | **Survey questions** | **Responses** |
| --- | --- | --- |
| **A** | **When reading information regarding vaccines provided by a health care professional, do you…** | 1= Never  2= Rarely  3= Sometimes  4= Often  5= Always |
| 1) | Find the print too small to read |  |
| 2) | Find characters and words that you do not understand |  |
| 3) | Find the text too hard to understand |  |
| 4) | Need a long time to read and understand the text |  |
| 5) | Need someone to help you read the information |  |
| **B** | **Since your child was born, you have…** | 1= Never  2= Rarely  3= Sometimes  4= Often  5= Always |
| 1) | Collected information on vaccinations from more than one source |  |
| 2) | Looked for information on the vaccines you were interested in |  |
| 3) | Understood the obtained information (on vaccines) |  |
| 4) | Discussed your thoughts about your child’s vaccinations with medical staff |  |
| 5) | Applied the obtained information to make decisions regarding your child’s vaccinations |  |
| **C** | **When your child needed or was recommended a vaccine, you have…** | 1= Never  2= Rarely  3= Sometimes  4= Often  5= Always |
| 1) | Considered the credibility of the information about the vaccines |  |
| 2) | Checked whether the information about the vaccines was valid and reliable |  |
| 3) | Looked for information that helped you make health-related decisions for your child |  |
| **D** | **When/after talking to a healthcare professional about vaccines for your child...** | 1= Never  2= Rarely  3= Sometimes  4= Often  5= Always |
| 1) | How often do you feel like you are able to give them all the information they need to help you |  |
| 2) | How often do you feel like you are able to ask all the questions you have |  |
| 3) | How often do you feel like you are able to make sure they explain anything that you do not understand |  |
| 4) | How likely are you to do your own background reading or gather additional information |  |
| 5) | How well do you think he or she listens to your questions and concerns about your child’s routine vaccinations |  |
| 6) | How often do they answer in a way that is easy for you to understand |  |
| 7) | How much influence do you feel you have on your child's vaccination schedule |  |
| **E** | **How much do you agree with each of the following statements?** | 1= Strongly disagree  2= Slightly disagree  3= Neutral  4= Slightly agree  5= Strongly agree |
| 1) | Healthcare professionals give out too many vaccines |  |
| 2) | Vaccines are a good way to protect my family/friends |  |
| 3) | I do not like the idea of vaccines for my child |  |
| 4) | Vaccines are generally safe |  |
| 5) | Vaccines can cause immediate, short-term side effects (such as fever, pain, etc.) |  |
| 6) | Vaccines are a way to take good care of my child now and in the future |  |
| 7) | My child is not afraid of shots/needles |  |
| 8) | Vaccines are effective |  |
| 9) | Vaccines contain dangerous ingredients |  |
| 10) | Vaccines can cause conditions such as autism or infertility |  |
| 11) | There is no need for my child to get vaccinated because everybody else does |  |
| 12) | Vaccines are important as they are beneficial to the community |  |
| 13) | I follow advice from friends/family/colleagues who think it is important to get vaccinated |  |
| 14) | I follow advice from friends/family/colleagues who think vaccination is **NOT** important, safe, or effective |  |
| 15) | I would prefer my child gain protection from an illness by catching the illness themselves rather than getting the vaccine |  |
| 16) | Vaccines can cause long-term side effects |  |
| 17) | Healthy children do not need vaccinations |  |
| 18) | I am more likely to trust vaccines that have been around longer, compared to newer vaccines |  |
| 19) | I trust science to develop safe and effective vaccines |  |
| 20) | I trust the government to ensure vaccines are safe and effective |  |
| 21) | Vaccination should be required for children to attend school |  |
| 22) | It is okay for the government to mandate vaccination |  |
| 23) | People should have the right to be medically exempt from receiving vaccines |  |
| 24) | People should have the right to claim religious exemptions from receiving vaccines |  |
| 25) | It is acceptable for companies/employers to require vaccines for employees to attend/return to work |  |
| 26) | People that are allergic to ingredients in the vaccine should have the right to be exempt from receiving vaccines |  |
| 27) | Children get more shots than are good for them |  |
| 28) | I believe that many of the illnesses that shots prevent are severe |  |
| 29) | It is better for my child to develop immunity by getting sick than to get a shot |  |
| 30) | It is better for children to get fewer vaccines at the same time |  |
| 31) | I trust the information I receive about shots. |  |
| 32) | I am able to openly discuss my concerns about shots with my child’s healthcare professional |  |
| **F** | **All things considered, how much do you trust your children's doctor?** | Scale from 1 to 10:  1= Do not trust at all  10= Completely trust |
| **G** | **Do you believe that children start receiving vaccines when they are too young?** | 1= Yes  2= No |
| **H** | **How concerned are you about each of the following items?** | 1= Very concerned  2= Somewhat concerned  3= Not sure  4= Not too concerned  5= Not at all concerned |
| 1) | Your child might have a serious side effect from a shot |  |
| 2) | Any one of the childhood shots might not be safe |  |
| 3) | A shot might not prevent disease |  |
| **I** | **Who benefits the MOST when you/children receive all of the recommended vaccines?** | 1= Yes  2= No |
| 1) | The child/myself |  |
| 2) | The community |  |
| 3) | The healthcare provider |  |
| 4) | The government |  |
| 5) | The vaccine/pharmaceutical companies |  |
| **J** | **How convenient is it for you to get to the following?** | 1= Not at all convenient  2= A little bit convenient  3= Somewhat convenient  4= Very convenient  5= Extremely convenient |
| 1) | Routine doctor’s visits (yearly visit, prescription renewal/ refills) |  |
| 2) | Non-routine doctor’s visits (sick or health concern visits) |  |
| 3) | Pharmacy |  |
| 4) | Specialists (not general or family practitioner) |  |
| 5) | Nearest hospital |  |
| 6) | Routine dental visits (i.e., cleanings) |  |
| 7) | Non-routine dental visits (i.e., toothache, fillings, root canals, other procedures) |  |

## Supplementary Material

### Supplementary Table S1. Associations of behaviors, beliefs, and experiences with parental vaccine literacy and hesitancy – unadjusted results

|  | Vaccine literacy (high vs. low) | |  | Vaccine hesitancy (yes vs. no) | |
| --- | --- | --- | --- | --- | --- |
|  | OR (95% CI) | P-value^A^ |  | OR (95% CI) | P-value^A^ |
| **Vaccine hesitancy** |  |  |  |  |  |
| Overall, how hesitant about childhood vaccines would you consider yourself to be? (1-5, very hesitant - not at all) | 0.98 (0.87, 1.11) | 0.751 |  | - |  |
| If you had another infant today, would you want him/her to get all the recommended shots? (yes/no) | 1.65 (1.03, 2.62) | **0.035** |  | - |  |
| **Vaccine literacy (1-5, not at all - extremely familiar)** |  |  |  |  |  |
| Familiarity with vaccines your child should receive | - |  |  | 0.85 (0.73, 1.00) | 0.051 |
| Familiarity with the schedule on your child's vaccines | - |  |  | 1.01 (0.87, 1.18) | 0.872 |
| **When reading information regarding vaccines provided by a health care professional, do you… (1-5, never - always)** |  |  |  |  |  |
| Find the print too small to read | 1.04 (0.92, 1.18) | 0.537 |  | 1.71 (1.51, 1.94) | **<0.001** |
| Find characters and words that you do not understand | 1.07 (0.94, 1.22) | 0.314 |  | 1.87 (1.65, 2.14) | **<0.001** |
| Find the text too hard to understand | 0.96 (0.85, 1.09) | 0.528 |  | 1.90 (1.67, 2.17) | **<0.001** |
| Need a long time to read and understand the text | 1.06 (0.93, 1.21) | 0.367 |  | 1.78 (1.57, 2.03) | **<0.001** |
| Need someone to help you read the information | 1.09 (0.96, 1.24) | 0.208 |  | 2.12 (1.85, 2.46) | **<0.001** |
| **Since your child was born, you have… (1-5, never - always)** |  |  |  |  |  |
| Collected information on vaccinations from more than one source | 1.71 (1.47, 2.00) | **<0.001** |  | 1.53 (1.34, 1.76) | **<0.001** |
| Looked for information on the vaccines you were interested in | 1.73 (1.50, 2.01) | **<0.001** |  | 1.49 (1.31, 1.71) | **<0.001** |
| Understood the obtained information (on vaccines) | 2.04 (1.71, 2.45) | **<0.001** |  | 1.03 (0.89, 1.20) | 0.691 |
| Discussed your thoughts about your child’s vaccinations with medical staff | 1.67 (1.44, 1.95) | **<0.001** |  | 1.13 (1.00, 1.29) | 0.060 |
| Applied the obtained information to make decisions regarding your child’s vaccinations | 1.73 (1.48, 2.04) | **<0.001** |  | 1.01 (0.88, 1.16) | 0.903 |
| **When your child needed or was recommended a vaccine, you have… (1-5, never - always)** |  |  |  |  |  |
| Considered the credibility of the information about the vaccines | 1.73 (1.49, 2.02) | **<0.001** |  | 1.20 (1.05, 1.37) | **0.008** |
| Checked whether the information about the vaccines was valid and reliable | 1.76 (1.52, 2.04) | **<0.001** |  | 1.35 (1.19, 1.54) | **<0.001** |
| Looked for information that helped you make health-related decisions for your child | 1.85 (1.58, 2.17) | **<0.001** |  | 1.32 (1.15, 1.51) | **<0.001** |
| **When/after talking to a healthcare professional about vaccines for your child... (1-5, never - always)** |  |  |  |  |  |
| How often do you feel like you are able to give them all the information they need to help you | 2.51 (2.03, 3.14) | **<0.001** |  | 0.68 (0.57, 0.81) | **<0.001** |
| How often do you feel like you are able to ask all the questions you have | 2.03 (1.68, 2.49) | **<0.001** |  | 0.80 (0.68, 0.94) | **0.009** |
| How often do you feel like you are able to make sure they explain anything that you do not understand | 2.08 (1.72, 2.53) | **<0.001** |  | 0.79 (0.67, 0.93) | **0.005** |
| How likely are you to do your own background reading or gather additional information | 1.68 (1.45, 1.95) | **<0.001** |  | 1.81 (1.57, 2.10) | **<0.001** |
| How well do you think he or she listens to your questions and concerns about your child’s routine vaccinations | 2.67 (2.16, 3.33) | **<0.001** |  | 1.08 (0.91, 1.28) | 0.397 |
| How often do they answer in a way that is easy for you to understand | 1.60 (1.38, 1.87) | **<0.001** |  | 0.77 (0.67, 0.88) | **<0.001** |
| How much influence do you feel you have on your child's vaccination schedule | 2.20 (1.86, 2.61) | **<0.001** |  | 0.90 (0.79, 1.03) | 0.141 |
| **How much do you agree with each of the following statements? (1-5, strongly disagree - strongly agree)** |  |  |  |  |  |
| Healthcare professionals give out too many vaccines | 1.04 (0.92, 1.18) | 0.507 |  | 2.35 (2.04, 2.72) | **<0.001** |
| Vaccines are a good way to protect my family/friends | 1.44 (1.20, 1.73) | **<0.001** |  | 0.34 (0.27, 0.42) | **<0.001** |
| I do not like the idea of vaccines for my child | 0.91 (0.81, 1.03) | 0.137 |  | 2.61 (2.26, 3.03) | **<0.001** |
| Vaccines are generally safe | 1.61 (1.34, 1.94) | **<0.001** |  | 0.44 (0.36, 0.53) | **<0.001** |
| Vaccines can cause immediate, short-term side effects (such as fever, pain, etc.) | 1.20 (1.01, 1.42) | **0.035** |  | 1.01 (0.87, 1.18) | 0.855 |
| Vaccines are a way to take good care of my child now and in the future | 1.44 (1.21, 1.72) | **<0.001** |  | 0.44 (0.36, 0.54) | **<0.001** |
| My child is not afraid of shots/needles | 1.30 (1.14, 1.49) | **<0.001** |  | 1.07 (0.96, 1.21) | 0.231 |
| Vaccines are effective | 1.61 (1.33, 1.94) | **<0.001** |  | 0.35 (0.27, 0.43) | **<0.001** |
| Vaccines contain dangerous ingredients | 0.96 (0.85, 1.10) | 0.586 |  | 2.52 (2.17, 2.94) | **<0.001** |
| Vaccines can cause conditions such as autism or infertility | 0.96 (0.85, 1.09) | 0.525 |  | 2.53 (2.19, 2.94) | **<0.001** |
| There is no need for my child to get vaccinated because everybody else does | 1.01 (0.90, 1.14) | 0.856 |  | 2.57 (2.23, 2.98) | **<0.001** |
| Vaccines are important as they are beneficial to the community | 1.45 (1.21, 1.73) | **<0.001** |  | 0.41 (0.34, 0.50) | **<0.001** |
| I follow advice from friends/family/colleagues who think it is important to get vaccinated | 1.32 (1.14, 1.53) | **<0.001** |  | 1.23 (1.08, 1.41) | **0.002** |
| I follow advice from friends/family/colleagues who think vaccination is NOT important, safe, or effective | 1.06 (0.94, 1.19) | 0.376 |  | 2.31 (2.02, 2.66) | **<0.001** |
| I would prefer my child gain protection from an illness by catching the illness themselves rather than getting the vaccine | 1.04 (0.92, 1.18) | 0.541 |  | 2.35 (2.05, 2.71) | **<0.001** |
| Vaccines can cause long-term side effects | 0.95 (0.83, 1.09) | 0.472 |  | 2.41 (2.07, 2.84) | **<0.001** |
| Healthy children do not need vaccinations | 0.97 (0.86, 1.09) | 0.590 |  | 2.58 (2.24, 2.99) | **<0.001** |
| I am more likely to trust vaccines that have been around longer, compared to newer vaccines | 1.43 (1.19, 1.72) | **<0.001** |  | 0.83 (0.70, 0.98) | **0.028** |
| I trust science to develop safe and effective vaccines | 1.49 (1.25, 1.77) | **<0.001** |  | 0.49 (0.40, 0.58) | **<0.001** |
| I trust the government to ensure vaccines are safe and effective | 1.30 (1.13, 1.50) | **<0.001** |  | 0.80 (0.71, 0.91) | **0.001** |
| Vaccination should be required for children to attend school | 1.36 (1.18, 1.56) | **<0.001** |  | 0.69 (0.60, 0.79) | **<0.001** |
| It is okay for the government to mandate vaccination | 1.25 (1.11, 1.40) | **<0.001** |  | 0.91 (0.82, 1.01) | 0.092 |
| People should have the right to be medically exempt from receiving vaccines | 1.15 (1.00, 1.33) | **0.049** |  | 1.68 (1.46, 1.94) | **<0.001** |
| People should have the right to claim religious exemptions from receiving vaccines | 1.09 (0.96, 1.24) | 0.190 |  | 1.93 (1.69, 2.21) | **<0.001** |
| It is acceptable for companies/employers to require vaccines for employees to attend/return to work | 1.28 (1.13, 1.45) | **<0.001** |  | 0.83 (0.74, 0.93) | **0.001** |
| People that are allergic to ingredients in the vaccine should have the right to be exempt from receiving vaccines | 1.37 (1.14, 1.65) | **0.001** |  | 0.94 (0.80, 1.11) | 0.491 |
| Children get more shots than are good for them | 1.06 (0.94, 1.21) | 0.342 |  | 2.80 (2.39, 3.31) | **<0.001** |
| I believe that many of the illnesses that shots prevent are severe | 1.19 (1.01, 1.41) | **0.037** |  | 0.64 (0.54, 0.75) | **<0.001** |
| It is better for my child to develop immunity by getting sick than to get a shot | 1.00 (0.88, 1.13) | 0.985 |  | 2.62 (2.27, 3.06) | **<0.001** |
| It is better for children to get fewer vaccines at the same time | 1.13 (0.97, 1.33) | 0.120 |  | 1.99 (1.70, 2.36) | **<0.001** |
| I trust the information I receive about shots | 1.31 (1.12, 1.52) | **0.001** |  | 0.48 (0.40, 0.57) | **<0.001** |
| I am able to openly discuss my concerns about shots with my child’s healthcare professional | 1.78 (1.49, 2.14) | **<0.001** |  | 0.50 (0.41, 0.60) | **<0.001** |
| How much do you trust your child's doctor (1-10) | 1.43 (1.28, 1.60) | **<0.001** |  | 0.70 (0.62, 0.79) | **<0.001** |
| Children start receiving vaccines when they are too young (yes/no) | 1.35 (0.93, 1.96) | 0.118 |  | 6.22 (4.40, 8.87) | **<0.001** |
| **How concerned are you about each of the following items? (1-5, very concerned- not at all concerned)** |  |  |  |  |  |
| Your child might have a serious side effect from a shot | 0.85 (0.74, 0.98) | **0.024** |  | 0.64 (0.56, 0.73) | **<0.001** |
| Any one of the childhood shots might not be safe | 0.91 (0.79, 1.04) | 0.169 |  | 0.44 (0.38, 0.51) | **<0.001** |
| A shot might not prevent disease | 0.84 (0.73, 0.97) | **0.014** |  | 0.44 (0.38, 0.51) | **<0.001** |
| **Who benefits the MOST when you/children receive all of the recommended vaccines? (yes/no)** |  |  |  |  |  |
| The child/myself | 0.87 (0.60, 1.24) | 0.448 |  | 0.26 (0.19, 0.36) | **<0.001** |
| The community | 1.47 (1.03, 2.12) | **0.036** |  | 0.34 (0.24, 0.46) | **<0.001** |
| The healthcare provider | 0.76 (0.52, 1.12) | 0.159 |  | 2.32 (1.63, 3.33) | **<0.001** |
| The government | 1.46 (0.86, 2.59) | 0.176 |  | 3.72 (2.28, 6.29) | **<0.001** |
| The vaccine/pharmaceutical companies | 1.32 (0.89, 2.00) | 0.178 |  | 2.23 (1.57, 3.18) | **<0.001** |
| **How convenient is it for you to get to the following? (1-5, not at all convenient - extremely convenient)** |  |  |  |  |  |
| Routine doctor’s visits (yearly visit, prescription renewal/ refills) | 1.70 (1.41, 2.06) | **<0.001** |  | 0.87 (0.73, 1.03) | 0.111 |
| Non-routine doctor’s visits (sick or health concern visits) | 1.39 (1.18, 1.64) | **<0.001** |  | 0.92 (0.80, 1.07) | 0.288 |
| Pharmacy | 1.73 (1.43, 2.10) | **<0.001** |  | 0.64 (0.54, 0.77) | **<0.001** |
| Specialists (not general or family practitioner) | 1.37 (1.16, 1.62) | **<0.001** |  | 1.13 (0.98, 1.31) | 0.101 |
| Nearest hospital | 1.48 (1.23, 1.79) | **<0.001** |  | 0.85 (0.72, 1.00) | 0.056 |
| Routine dental visits (i.e., cleanings) | 1.50 (1.24, 1.81) | **<0.001** |  | 0.80 (0.67, 0.95) | **0.011** |
| Non-routine dental visits (i.e., toothache, fillings, root canals, other procedures) | 1.43 (1.21, 1.70) | **<0.001** |  | 1.08 (0.93, 1.25) | 0.337 |

CI, confidence interval; OR, odds ratio

^A^ P-values in bold indicate statistical significance.

### Supplementary Table S2. Derivation of factors from survey questions^A^

|  | Factor1 | Factor2 | Factor3 | Factor4 | Factor5 | Factor6 | Factor7 |
| --- | --- | --- | --- | --- | --- | --- | --- |
| **When reading information regarding vaccines provided by a health care professional, do you… (1-5, never - always)** |  |  |  |  |  |  |  |
| Find the print too small to read | -0.021 | 0.029 | -0.015 | **0.810** | 0.001 | 0.020 | -0.067 |
| Find characters and words that you do not understand | 0.012 | 0.014 | 0.076 | **0.800** | -0.027 | 0.034 | -0.015 |
| Find the text too hard to understand | 0.088 | 0.010 | -0.037 | **0.889** | 0.036 | 0.032 | 0.030 |
| Need a long time to read and understand the text | 0.020 | -0.017 | -0.032 | **0.852** | 0.069 | -0.004 | -0.013 |
| Need someone to help you read the information | 0.194 | 0.082 | 0.042 | **0.715** | 0.012 | -0.083 | 0.006 |
| **Since your child was born, you have… (1-5, never - always)** |  |  |  |  |  |  |  |
| Collected information on vaccinations from more than one source | 0.032 | 0.025 | **0.776** | 0.069 | -0.069 | -0.060 | -0.012 |
| Looked for information on the vaccines you were interested in | -0.036 | 0.025 | **0.870** | 0.074 | -0.146 | -0.069 | 0.012 |
| Understood the obtained information (on vaccines) | 0.071 | 0.034 | **0.412** | -0.018 | **0.337** | 0.005 | 0.111 |
| Discussed your thoughts about your child’s vaccinations with medical staff | -0.064 | -0.034 | **0.628** | 0.071 | 0.143 | -0.009 | 0.000 |
| Applied the obtained information to make decisions regarding your child’s vaccinations | -0.035 | -0.050 | **0.617** | 0.025 | 0.220 | 0.020 | 0.116 |
| **When your child needed or was recommended a vaccine, you have… (1-5, never - always)** |  |  |  |  |  |  |  |
| Considered the credibility of the information about the vaccines | -0.040 | -0.060 | **0.774** | -0.010 | 0.025 | 0.106 | 0.092 |
| Checked whether the information about the vaccines was valid and reliable | -0.041 | -0.068 | **0.880** | -0.058 | -0.021 | 0.026 | 0.026 |
| Looked for information that helped you make health-related decisions for your child | -0.048 | -0.033 | **0.903** | -0.062 | -0.086 | 0.066 | -0.002 |
| **When/after talking to a healthcare professional about vaccines for your child... (1-5, never - always)** |  |  |  |  |  |  |  |
| How often do you feel like you are able to give them all the information they need to help you | 0.010 | -0.007 | -0.061 | -0.007 | **0.804** | -0.011 | -0.027 |
| How often do you feel like you are able to ask all the questions you have | 0.011 | -0.131 | -0.081 | 0.034 | **0.878** | -0.037 | -0.025 |
| How often do you feel like you are able to make sure they explain anything that you do not understand | 0.002 | -0.068 | -0.033 | 0.060 | **0.829** | -0.011 | -0.028 |
| How likely are you to do your own background reading or gather additional information | 0.076 | -0.018 | **0.601** | -0.066 | -0.059 | -0.013 | -0.242 |
| How well do you think he or she listens to your questions and concerns about your child’s routine vaccinations | 0.087 | 0.171 | 0.079 | -0.010 | **0.455** | -0.016 | -0.100 |
| How often do they answer in a way that is easy for you to understand | -0.065 | 0.127 | -0.027 | -0.072 | **0.330** | 0.061 | -0.073 |
| How much influence do you feel you have on your child's vaccination schedule | -0.009 | 0.037 | 0.097 | -0.057 | 0.260 | 0.101 | -0.054 |
| **How much do you agree with each of the following statements? (1-5, strongly disagree - strongly agree)** |  |  |  |  |  |  |  |
| Healthcare professionals give out too many vaccines | **0.714** | -0.002 | -0.011 | -0.049 | -0.048 | 0.117 | -0.134 |
| Vaccines are a good way to protect my family/friends | -0.211 | **0.696** | -0.009 | 0.034 | -0.084 | 0.187 | 0.047 |
| I do not like the idea of vaccines for my child | **0.882** | -0.121 | -0.022 | 0.000 | 0.030 | 0.042 | 0.133 |
| Vaccines are generally safe | -0.086 | **0.773** | -0.049 | 0.012 | -0.035 | 0.060 | 0.068 |
| Vaccines can cause immediate, short-term side effects (such as fever, pain, etc.) | 0.216 | 0.110 | -0.097 | 0.012 | 0.106 | **0.373** | 0.046 |
| Vaccines are a way to take good care of my child now and in the future | -0.188 | **0.703** | -0.053 | 0.046 | -0.028 | 0.159 | -0.002 |
| My child is not afraid of shots/needles | **0.414** | 0.274 | -0.026 | -0.076 | 0.084 | -0.057 | 0.115 |
| Vaccines are effective | -0.189 | **0.737** | 0.014 | 0.022 | -0.096 | 0.159 | 0.064 |
| Vaccines contain dangerous ingredients | **0.734** | -0.063 | -0.123 | -0.008 | 0.042 | 0.124 | -0.097 |
| Vaccines can cause conditions such as autism or infertility | **0.762** | -0.043 | -0.082 | 0.009 | 0.064 | 0.034 | -0.083 |
| There is no need for my child to get vaccinated because everybody else does | **0.899** | -0.020 | -0.019 | 0.028 | 0.055 | -0.033 | 0.138 |
| Vaccines are important as they are beneficial to the community | -0.195 | **0.653** | -0.053 | 0.082 | 0.010 | 0.110 | 0.008 |
| I follow advice from friends/family/colleagues who think it is important to get vaccinated | 0.287 | 0.509 | 0.212 | -0.021 | -0.167 | -0.074 | -0.013 |
| I follow advice from friends/family/colleagues who think vaccination is NOT important, safe, or effective | **0.793** | 0.087 | 0.030 | 0.045 | -0.075 | -0.027 | 0.081 |
| I would prefer my child gain protection from an illness by catching the illness themselves rather than getting the vaccine | **0.752** | -0.043 | -0.015 | 0.014 | 0.039 | 0.012 | -0.010 |
| Vaccines can cause long-term side effects | **0.667** | -0.042 | -0.035 | -0.055 | -0.007 | 0.191 | -0.086 |
| Healthy children do not need vaccinations | **0.924** | -0.006 | -0.007 | 0.019 | 0.044 | -0.067 | 0.132 |
| I am more likely to trust vaccines that have been around longer, compared to newer vaccines | 0.017 | 0.204 | 0.047 | 0.013 | -0.113 | **0.506** | -0.078 |
| I trust science to develop safe and effective vaccines | 0.019 | **0.810** | -0.027 | -0.034 | -0.034 | -0.042 | 0.062 |
| I trust the government to ensure vaccines are safe and effective | 0.176 | **0.781** | 0.001 | 0.013 | -0.032 | -0.263 | 0.065 |
| Vaccination should be required for children to attend school | 0.020 | **0.784** | -0.062 | 0.054 | 0.055 | **-0.382** | -0.058 |
| It is okay for the government to mandate vaccination | 0.206 | **0.844** | -0.013 | -0.003 | -0.022 | **-0.595** | -0.113 |
| People should have the right to be medically exempt from receiving vaccines | **0.402** | -0.099 | 0.064 | -0.017 | -0.087 | **0.544** | 0.037 |
| People should have the right to claim religious exemptions from receiving vaccines | **0.446** | -0.168 | 0.035 | 0.038 | -0.029 | **0.427** | -0.007 |
| It is acceptable for companies/employers to require vaccines for employees to attend/return to work | 0.148 | **0.846** | 0.035 | -0.037 | -0.018 | **-0.523** | -0.057 |
| People that are allergic to ingredients in the vaccine should have the right to be exempt from receiving vaccines | 0.115 | -0.032 | -0.018 | -0.008 | 0.085 | **0.540** | 0.050 |
| Children get more shots than are good for them | **0.564** | 0.006 | 0.099 | 0.036 | -0.103 | 0.068 | -0.148 |
| I believe that many of the illnesses that shots prevent are severe | -0.084 | **0.478** | -0.047 | 0.050 | 0.013 | 0.190 | 0.000 |
| It is better for my child to develop immunity by getting sick than to get a shot | **0.688** | -0.006 | 0.013 | 0.017 | -0.081 | 0.059 | -0.121 |
| It is better for children to get fewer vaccines at the same time | **0.342** | 0.032 | 0.048 | 0.049 | -0.012 | 0.220 | -0.141 |
| I trust the information I receive about shots | -0.099 | **0.560** | 0.045 | -0.135 | 0.037 | -0.018 | -0.005 |
| I am able to openly discuss my concerns about shots with my child’s healthcare professional | -0.132 | **0.389** | 0.016 | -0.110 | 0.135 | 0.101 | -0.067 |
| How much do you trust your child's doctor (1-10) | -0.025 | **0.457** | 0.026 | 0.003 | 0.201 | -0.021 | 0.012 |
| **How concerned are you about each of the following items? (1-5, very concerned- not at all concerned)** |  |  |  |  |  |  |  |
| Your child might have a serious side effect from a shot | 0.075 | 0.061 | -0.082 | 0.018 | 0.018 | -0.014 | **0.560** |
| Any one of the childhood shots might not be safe | -0.054 | 0.050 | 0.034 | 0.000 | -0.009 | 0.035 | **0.798** |
| A shot might not prevent disease | -0.021 | 0.018 | 0.065 | -0.036 | -0.082 | 0.080 | **0.835** |

^A^ This analysis included all questions in Table 2, except those about convenience, who benefits the most, and whether children start receiving vaccines when they are too young. Correlations were tested between each pair of included variables, and variables with a loading value >±0.3 were considered significant contributors to a factor and are shown in bold.

### Supplementary Table S3. Association of factors with vaccine literacy and hesitancy – unadjusted results

|  | Vaccine literacy (high vs. low) | |  | Vaccine hesitancy (yes vs. no) | |
| --- | --- | --- | --- | --- | --- |
| Factor | OR (95% CI) | P-value^A^ |  | OR (95% CI) | P-value^A^ |
| 1: Negative beliefs about vaccines | 0.99 (0.83, 1.17) | 0.877 |  | 3.53 (2.88, 4.38) | **<0.001** |
| 2: Positive beliefs about vaccines | 1.42 (1.20, 1.68) | **<0.001** |  | 0.56 (0.47, 0.66) | **<0.001** |
| 3: Active information-seeking behavior | 1.95 (1.64, 2.34) | **<0.001** |  | 1.34 (1.15, 1.56) | **<0.001** |
| 4: Trouble understanding information from HCPs | 0.91 (0.76, 1.08) | 0.264 |  | 1.62 (1.39, 1.91) | **<0.001** |
| 5: Positive interactions with HCPs | 2.18 (1.80, 2.66) | **<0.001** |  | 0.82 (0.70, 0.95) | **0.010** |
| 6: Beliefs about vaccination requirements | 1.15 (0.97, 1.37) | 0.105 |  | 0.83 (0.72, 0.97) | **0.018** |
| 7: Concerns about children’s vaccines | 0.82 (0.69, 0.98) | **0.026** |  | 0.51 (0.43, 0.60) | **<0.001** |

CI, confidence interval; HCP, healthcare provider; OR, odds ratio

^A^ P-values in bold indicate statistical significance.

### Supplementary Table S4. Pairwise associations of literacy regarding specific vaccine-preventable diseases

|  | **Chickenpox/ varicella** | **COVID-19** | **Diphtheria** | **Influenza** | **Hemophilus influenza** | **Hepatitis A** | **Hepatitis B** | **Human papillomavirus** | **Measles** | **Meningitis** | **Mumps** | **Pertussis/whooping cough** | **Polio** | **Pneumococcal/pneumonia** | **Rotavirus** | **Rubella** | **Tetanus** |
| --- | --- | --- | --- | --- | --- | --- | --- | --- | --- | --- | --- | --- | --- | --- | --- | --- | --- |
| **Chickenpox/varicella** | 1.000 | **<0.001** | **<0.001** | **<0.001** | **<0.001** | **<0.001** | **<0.001** | **<0.001** | **0.007** | **<0.001** | **<0.001** | **<0.001** | **0.001** | **0.016** | **<0.001** | **<0.001** | 0.140 |
| **COVID-19** | **<0.001** | 1.000 | **<0.001** | 1.000 | **<0.001** | **<0.001** | **<0.001** | **<0.001** | **<0.001** | **<0.001** | **<0.001** | **<0.001** | **<0.001** | **<0.001** | **<0.001** | **<0.001** | **<0.001** |
| **Diphtheria** | **<0.001** | **<0.001** | 1.000 | **<0.001** | **0.002** | 0.259 | 0.068 | **0.003** | **0.000** | **0.004** | **0.006** | **0.024** | **0.000** | **0.000** | **0.017** | 1.000 | **0.000** |
| **Influenza** | **<0.001** | 1.000 | **<0.001** | 1.000 | **<0.001** | **<0.001** | **<0.001** | **<0.001** | **<0.001** | **<0.001** | **<0.001** | **<0.001** | **<0.001** | **<0.001** | **<0.001** | **<0.001** | **<0.001** |
| **Hemophilus influenza** | **<0.001** | **<0.001** | **0.002** | **<0.001** | 1.000 | **<0.001** | **<0.001** | **<0.001** | **<0.001** | **<0.001** | **<0.001** | **<0.001** | **<0.001** | **<0.001** | 0.512 | **0.002** | **<0.001** |
| **Hepatitis A** | **<0.001** | **<0.001** | 0.259 | **<0.001** | **<0.001** | 1.000 | 0.518 | 0.067 | **0.001** | 0.095 | 0.118 | 0.281 | **0.003** | **<0.001** | **<0.001** | 0.237 | **<0.001** |
| **Hepatitis B** | **<0.001** | **<0.001** | 0.068 | **<0.001** | **<0.001** | 0.518 | 1.000 | 0.257 | **0.006** | 0.331 | 0.388 | 0.706 | 0.023 | **0.002** | **<0.001** | 0.060 | **<0.001** |
| **Human papillomavirus** | **<0.001** | **<0.001** | **0.003** | **<0.001** | **<0.001** | 0.067 | 0.257 | 1.000 | 0.113 | 0.914 | 0.828 | 0.482 | 0.276 | 0.063 | **<0.001** | **0.002** | **0.005** |
| **Measles** | **0.007** | **<0.001** | **<0.001** | **<0.001** | **<0.001** | **0.001** | **0.006** | 0.113 | 1.000 | 0.081 | 0.064 | **0.019** | 0.660 | 0.825 | **<0.001** | **<0.001** | 0.244 |
| **Meningitis** | **<0.001** | **<0.001** | **0.004** | **<0.001** | **<0.001** | 0.095 | 0.331 | 0.914 | 0.081 | 1.000 | 0.957 | 0.589 | 0.211 | **0.043** | **<0.001** | **0.004** | **0.003** |
| **Mumps** | **<0.001** | **<0.001** | **0.006** | **<0.001** | **<0.001** | 0.118 | 0.388 | 0.828 | 0.064 | 0.957 | 1.000 | 0.665 | 0.174 | **0.033** | **<0.001** | **0.005** | **0.002** |
| **Pertussis/whooping cough** | **<0.001** | **<0.001** | **0.024** | **<0.001** | **<0.001** | 0.281 | 0.706 | 0.482 | **0.019** | 0.589 | 0.665 | 1.000 | 0.065 | **0.009** | **<0.001** | **0.021** | **<0.001** |
| **Polio** | **0.001** | **<0.001** | **<0.001** | **<0.001** | **<0.001** | **0.003** | **0.023** | 0.276 | 0.660 | 0.211 | 0.174 | 0.065 | 1.000 | 0.475 | **<0.001** | **<0.001** | 0.097 |
| **Pneumococcal/pneumonia** | **0.016** | **<0.001** | **<0.001** | **<0.001** | **<0.001** | **<0.001** | **0.002** | 0.063 | 0.825 | **0.043** | **0.033** | **0.009** | 0.475 | 1.000 | **<0.001** | **<0.001** | 0.374 |
| **Rotavirus** | **<0.001** | **<0.001** | **0.017** | **<0.001** | 0.512 | **<0.001** | **<0.001** | **<0.001** | **<0.001** | **<0.001** | **<0.001** | **<0.001** | **<0.001** | **<0.001** | 1.000 | **0.020** | **<0.001** |
| **Rubella** | **<0.001** | **<0.001** | 1.000 | **<0.001** | **0.002** | 0.237 | 0.060 | **0.002** | **<0.001** | **0.004** | **0.005** | **0.021** | **<0.001** | **<0.001** | **0.020** | 1.000 | **<0.001** |
| **Tetanus** | 0.140 | **<0.001** | **<0.001** | **<0.001** | **<0.001** | **<0.001** | **<0.001** | **0.005** | 0.244 | **0.003** | **0.002** | **<0.001** | 0.097 | 0.374 | **<0.001** | **<0.001** | 1.000 |

The table presents the P-values for chi-square tests of literacy for each pair of diseases. P-values <0.05 are shown in bold, and indicate that high literacy regarding the first disease was correlated with high literacy regarding the second disease.

### Supplementary Table S5. Pairwise associations of hesitancy regarding specific vaccine-preventable diseases

|  | **Chickenpox/ varicella** | **COVID-19** | **Diphtheria** | **Influenza** | **Hemophilus influenza** | **Hepatitis A** | **Hepatitis B** | **Human papillomavirus** | **Measles** | **Meningitis** | **Mumps** | **Pertussis/whooping cough** | **Polio** | **Pneumococcal/pneumonia** | **Rotavirus** | **Rubella** | **Tetanus** |
| --- | --- | --- | --- | --- | --- | --- | --- | --- | --- | --- | --- | --- | --- | --- | --- | --- | --- |
| **Chickenpox/varicella** | 1.000 | **<0.001** | 0.400 | **<0.001** | 0.034 | 0.341 | 0.919 | 0.679 | 0.604 | 0.341 | 0.604 | 0.400 | 0.034 | 0.531 | 0.438 | 0.604 | 0.400 |
| **COVID-19** | **<0.001** | 1.000 | **<0.001** | 0.786 | **<0.001** | **<0.001** | **<0.001** | **<0.001** | **<0.001** | **<0.001** | **<0.001** | **<0.001** | **<0.001** | **<0.001** | **<0.001** | **<0.001** | **<0.001** |
| **Diphtheria** | 0.400 | **<0.001** | 1.000 | **<0.001** | 0.241 | 1.000 | 0.524 | 0.746 | 0.828 | 1.000 | 0.828 | 1.000 | 0.241 | 0.913 | 1.000 | 0.828 | 1.000 |
| **Influenza** | **<0.001** | 0.786 | **<0.001** | 1.000 | **<0.001** | **<0.001** | **<0.001** | **<0.001** | **<0.001** | **<0.001** | **<0.001** | **<0.001** | **<0.001** | **<0.001** | **<0.001** | **<0.001** | **<0.001** |
| **Hemophilus influenza** | 0.034 | **<0.001** | 0.241 | **<0.001** | 1.000 | 0.288 | 0.056 | 0.109 | 0.134 | 0.288 | 0.134 | 0.241 | 1.000 | 0.164 | 0.218 | 0.134 | 0.241 |
| **Hepatitis A** | 0.341 | **<0.001** | 1.000 | **<0.001** | 0.288 | 1.000 | 0.455 | 0.664 | 0.744 | 1.000 | 0.744 | 1.000 | 0.288 | 0.827 | 0.950 | 0.744 | 1.000 |
| **Hepatitis B** | 0.919 | **<0.001** | 0.524 | **<0.001** | 0.056 | 0.455 | 1.000 | 0.835 | 0.753 | 0.455 | 0.753 | 0.524 | 0.056 | 0.674 | 0.567 | 0.753 | 0.524 |
| **Human papillomavirus** | 0.679 | **<0.001** | 0.746 | **<0.001** | 0.109 | 0.664 | 0.835 | 1.000 | 1.000 | 0.664 | 1.000 | 0.746 | 0.109 | 0.915 | 0.796 | 1.000 | 0.746 |
| **Measles** | 0.604 | **<0.001** | 0.828 | **<0.001** | 0.134 | 0.744 | 0.753 | 1.000 | 1.000 | 0.744 | 1.000 | 0.828 | 0.134 | 1.000 | 0.879 | 1.000 | 0.828 |
| **Meningitis** | 0.341 | **<0.001** | 1.000 | **<0.001** | 0.288 | 1.000 | 0.455 | 0.664 | 0.744 | 1.000 | 0.744 | 1.000 | 0.288 | 0.827 | 0.950 | 0.744 | 1.000 |
| **Mumps** | 0.604 | **<0.001** | 0.828 | **<0.001** | 0.134 | 0.744 | 0.753 | 1.000 | 1.000 | 0.744 | 1.000 | 0.828 | 0.134 | 1.000 | 0.879 | 1.000 | 0.828 |
| **Pertussis/whooping cough** | 0.400 | **<0.001** | 1.000 | **<0.001** | 0.241 | 1.000 | 0.524 | 0.746 | 0.828 | 1.000 | 0.828 | 1.000 | 0.241 | 0.913 | 1.000 | 0.828 | 1.000 |
| **Polio** | 0.034 | **<0.001** | 0.241 | **<0.001** | 1.000 | 0.288 | 0.056 | 0.109 | 0.134 | 0.288 | 0.134 | 0.241 | 1.000 | 0.164 | 0.218 | 0.134 | 0.241 |
| **Pneumococcal/pneumonia** | 0.531 | **<0.001** | 0.913 | **<0.001** | 0.164 | 0.827 | 0.674 | 0.915 | 1.000 | 0.827 | 1.000 | 0.913 | 0.164 | 1.000 | 0.964 | 1.000 | 0.913 |
| **Rotavirus** | 0.438 | **<0.001** | 1.000 | **<0.001** | 0.218 | 0.950 | 0.567 | 0.796 | 0.879 | 0.950 | 0.879 | 1.000 | 0.218 | 0.964 | 1.000 | 0.879 | 1.000 |
| **Rubella** | 0.604 | **<0.001** | 0.828 | **<0.001** | 0.134 | 0.744 | 0.753 | 1.000 | 1.000 | 0.744 | 1.000 | 0.828 | 0.134 | 1.000 | 0.879 | 1.000 | 0.828 |
| **Tetanus** | 0.400 | **<0.001** | 1.000 | **<0.001** | 0.241 | 1.000 | 0.524 | 0.746 | 0.828 | 1.000 | 0.828 | 1.000 | 0.241 | 0.913 | 1.000 | 0.828 | 1.000 |

The table presents the P-values for chi-square tests of hesitancy for each pair of diseases. P-values <0.05 are shown in bold, and indicate that hesitancy regarding the first disease was correlated with hesitancy regarding the second disease.
